# Lack of strong innate immune reactivity renders macrophages alone unable to control productive Varicella-Zoster Virus infection in an isogenic human iPSC-derived neuronal co-culture model

**DOI:** 10.3389/fimmu.2023.1177245

**Published:** 2023-05-23

**Authors:** Elise Van Breedam, Tamariche Buyle-Huybrecht, Jonas Govaerts, Pieter Meysman, Andrea Bours, Marlies Boeren, Julia Di Stefano, Thalissa Caers, Hans De Reu, Laura Dirkx, Jolien Schippers, Esther Bartholomeus, Marielle Lebrun, Catherine Sadzot-Delvaux, Paulina Rybakowska, Marta E. Alarcón-Riquelme, Concepción Marañón, Kris Laukens, Peter Delputte, Benson Ogunjimi, Peter Ponsaerts

**Affiliations:** ^1^ Laboratory of Experimental Hematology (LEH), Vaccine and Infectious Disease Institute (VAXINFECTIO), University of Antwerp, Antwerp, Belgium; ^2^ Antwerp Center for Translational Immunology and Virology (ACTIV), Vaccine and Infectious Disease Institute (VAXINFECTIO), University of Antwerp, Antwerp, Belgium; ^3^ Laboratory of Microbiology, Parasitology and Hygiene (LMPH), University of Antwerp, Antwerp, Belgium; ^4^ Antwerp Unit for Data Analysis and Computation in Immunology and Sequencing (AUDACIS), University of Antwerp, Antwerp, Belgium; ^5^ Adrem Data Lab, Department of Computer Science, University of Antwerp, Antwerp, Belgium; ^6^ Biomedical Informatics Research Network Antwerp (Biomina), University of Antwerp, Antwerp, Belgium; ^7^ Flow Cytometry and Cell Sorting Core Facility (FACSUA), Laboratory of Experimental Hematology (LEH), Vaccine and Infectious Disease Institute (VAXINFECTIO), University of Antwerp, Antwerp, Belgium; ^8^ Laboratory of Virology and Immunology, Interdisciplinary Research Institute in the Biomedical Sciences GIGA-Infection, Inflammation and Immunity, University of Liège, Liège, Belgium; ^9^ Department of Genomic Medicine, Centre for Genomics and Oncological Research (GENYO), Pfizer-University of Granada-Junta de Andalucía, Parque Tecnológico de la Salud (PTS), Granada, Spain; ^10^ Infla-Med, University of Antwerp, Antwerp, Belgium; ^11^ Centre for Health Economics Research & Modelling Infectious Diseases (CHERMID), Vaccine & Infectious Disease Institute (VAXINFECTIO), University of Antwerp, Antwerp, Belgium; ^12^ Department of Paediatrics, Antwerp University Hospital, Antwerp, Belgium

**Keywords:** varicella zoster virus, iPSC, neurons, macrophages, neuronal models, axonal infection, innate immune response, RNA-seq analysis

## Abstract

With Varicella-Zoster Virus (VZV) being an exclusive human pathogen, human induced pluripotent stem cell (hiPSC)-derived neural cell culture models are an emerging tool to investigate VZV neuro-immune interactions. Using a compartmentalized hiPSC-derived neuronal model allowing axonal VZV infection, we previously demonstrated that paracrine interferon (IFN)-α2 signalling is required to activate a broad spectrum of interferon-stimulated genes able to counteract a productive VZV infection in hiPSC-neurons. In this new study, we now investigated whether innate immune signalling by VZV-challenged macrophages was able to orchestrate an antiviral immune response in VZV-infected hiPSC-neurons. In order to establish an isogenic hiPSC-neuron/hiPSC-macrophage co-culture model, hiPSC-macrophages were generated and characterised for phenotype, gene expression, cytokine production and phagocytic capacity. Even though immunological competence of hiPSC-macrophages was shown following stimulation with the poly(dA:dT) or treatment with IFN-α2, hiPSC-macrophages in co-culture with VZV-infected hiPSC-neurons were unable to mount an antiviral immune response capable of suppressing a productive neuronal VZV infection. Subsequently, a comprehensive RNA-Seq analysis confirmed the lack of strong immune responsiveness by hiPSC-neurons and hiPSC-macrophages upon, respectively, VZV infection or challenge. This may suggest the need of other cell types, like T-cells or other innate immune cells, to (co-)orchestrate an efficient antiviral immune response against VZV-infected neurons.

## Importance

VZV is a neurotropic herpesvirus that naturally infects over 95% of the human population, resulting in a large burden of disease. *In vitro* human iPSC-derived models that mimic VZV’s entry route into the nervous system via axon termini, and additionally allow interaction with immune cell populations, can progress our understanding of innate immune cell responses to neuronal VZV infection and replication. Here we demonstrate that hiPSC-macrophages, even though unable to control an ongoing VZV infection in hiPSC-neurons on their own, are – in contrast to hiPSC-neurons – not ignorant towards VZV challenge. Therefore, we believe our established bi-partite hiPSC-derived neuro-immune co-culture model for VZV infection to be highly suitable for furthers studies aiming to link innate and adaptive immunity towards VZV using even more advanced iPSC-derived neuro-immune co-culture models.

## Introduction

Varicella-zoster virus (VZV) or human herpesvirus 3 (HHV-3) is a highly species-specific human neurotropic alpha herpesvirus. Upon initial infection - typically during childhood years - the virus enters the host via the upper respiratory mucosal epithelium, which serves as entry point for the initial VZV spread to local lymphoid tissues. Here, VZV infects T-cells, which then act as vehicle to spread the virus via the blood to the skin, leading to the initial distinct VZV pathology known as varicella or chickenpox (1). This first viraemic phase of VZV is eventually controlled by both innate and adaptive immune responses. The innate immune system is believed to rely mainly on the type-I interferon (IFN) response and circulating innate immune cells, such as NK-cells, monocytes/macrophages and dendritic cells, while CD4^+^ T-cells are of central importance during the subsequent adaptive immune response (2–6). Although the host immune system can control productive VZV infection, the virus remains present in a latent state in neurons of the sensory ganglia. Upon reactivation from latency, which is typically associated with weakened host immunity, VZV travels to the skin and cause herpes zoster (HZ), a more localized, usually unilateral, painful skin rash with blisters. In addition, around 20% of HZ patients suffers from long-lasting pain known as postherpetic neuralgia (7). Furthermore, VZV infection may also lead to severe complications, including encephalitis, cerebellitis and CNS vasculopathy (2).

A typical hallmark of VZV infections is that the interaction between VZV and neurons is of central importance and markedly distinct from the interaction with other cell types. Indeed, previous studies indicated that VZV infection of neurons is less severe than in non-neuronal cells and that VZV-infected neurons are more resistant to apoptosis, albeit results may depend on the actual cell types and/or VZV strain used (8). Nevertheless, many key aspects of the VZV-neural cell interaction remain incompletely understood. Unfortunately, the human-tropic nature of VZV and the lack of small animal models able to evoke a response to VZV that is fully equivalent to that in humans complicate our understanding of its neurobiology (5, 9). *In vitro*, the scarce availability and inherent variability of primary human neural tissue have been hampering progress in VZV research. In the last decade, neuronal *in vitro* models derived from human embryonic stem cells (hESC) and human induced pluripotent stem cells (hiPSC) have become important platforms in the study of VZV neurobiology (10–13). Furthermore, these hESC- or hiPSC-derived neurons can be cultured in compartmentalized chambers that separate the axons from the neuronal cell bodies. This model then allows for axonal VZV infection, mimicking the natural infection route (13, 14). In addition, the differentiation capabilities of pluripotent stem cells provide the potential to create multicellular autologous neuro-immune co-culture systems, in which more complex virus-host interactions can be studied.

In a previous study by our group, a compartmentalized hiPSC-derived neuronal model of VZV infection was developed to evaluate neuronal type-I IFN response following VZV infection (9). Although Sen and colleagues point to the superior potency of the type-II IFN response (IFN-γ) in the control of VZV infection (15), both experimental and clinical observations also illustrate the importance of the type-I IFN response (IFN-α/β) during the early infection stages (6, 16). In fact, many immunodeficiencies associated with severe VZV infections affect type-I IFN signaling pathways (16, 17). We showed that while hiPSC-neurons adequately respond to exogenous IFN-α treatment by the production of interferon stimulated genes (ISGs) and the reduction of VZV replication and spread, they do not produce detectable levels of IFN-α2 or upregulate ISGs by themselves upon infection with VZV (9). We therefore hypothesized the potential role of circulating innate immune cells in the production of type-I IFNs that could limit neuronal VZV infection. In this study, we specifically focus on the role of macrophages in the control of a productive neuronal VZV infection. To this end, the compartmentalized neuronal model was extended to a di-cellular neuro-immune co-culture model by including autologous hiPSC-macrophages in the cell body compartment. Although several groups have studied VZV infection in peripheral blood mononuclear cells (PBMCs) and subsets of PBMCs before (17–21), few have focused on monocytes and macrophages in particular (3, 4). Kennedy demonstrated the permissiveness of monocytes to productive VZV infection (4) and several other groups described innate immune activation in monocytes following VZV infection in both experimental and clinical observations (22–24). The contribution of macrophages to the innate immune response against a neuronal VZV infection however requires further investigation.

## Materials and methods

### Differentiation of hiPSC-neurons

A previously established hiPSC-derived neural stem cell line (hiPSC-NSC) (25) was differentiated to obtain Tuj1^+^ NeuN^+^ peripherin^+^ neurons, according to the protocol described in our earlier work (9). In brief, expanded hiPSC-NSC were grown onto poly-L-ornithine (20 µg/ml, Sigma) and laminin (10 µg/ml, Sigma)-coated well plates in neuronal differentiation medium (Neurobasal Plus medium (Gibco), 1 × B27 plus supplement (Gibco), 2 mM L-glutamine (Gibco), 1 × CultureOne supplement (Gibco), 200 µM ascorbic acid (Sigma), 1% PS (Gibco), 10 ng/ml recombinant human brain-derived neurotrophic factor (rhBDNF) and recombinant human glial cell line-derived neurotrophic factor (rhGDNF) (Immunotools)) for a minimum of 21 days. Medium was changed every 2-3 days. Cultures were maintained at 37°C and 5% CO_2_ throughout the entire protocol. Brightfield images of hiPSC-neurons were obtained using a Fluovert Leitz microscope equipped with an Olympus UC30 camera.

### Differentiation of hiPSC-macrophages

hiPSC – the original hiPSC line from which hiPSC-NSC were derived (25) – were first differentiated into hematopoietic progenitor cells (hiPSC-HPC) using the STEMdiff Hematopoietic kit (STEMCELL Technologies) in 12 days, according to manufacturer’s instructions. At day 12, generated hiPSC-HPC were harvested and resuspended at a concentration of 3-5 x 10^5^ cells/ml in StemSpan SFEMII medium (STEMCELL Technologies) containing 100 ng/ml SCF Immunotools), 100 ng/ml TPO (PeproTech), 100 ng/ml Flt-3l (Immunotools), 50 ng/ml M-CSF (Immunotools) and 20 ng/ml GM-CSF (Immunotools) to allow expansion of the cells. After 2 days of expansion (day 14), cells were passaged at a concentration of 3-5 x 10^5^ cells/ml and allowed to grow for another 3 days. At day 17, expanded hiPSC-HPC were harvested and resuspended at a concentration of 0.5 x 10^6^ cells/ml in X-Vivo medium (Lonza) supplemented with 100 ng/ml M-CSF (Immunotools) to direct further differentiation into hiPSC-macrophages. Brightfield images of hiPSC-macrophages were obtained using a Fluovert Leitz microscope equipped with an Olympus UC30 camera. Finally, at day 24, hiPSC-macrophages were harvested and subjected to a CD14 selection step using anti-human CD14 magnetic beads (Miltenyi Biotec) for downstream experiments. Note that no selection step was performed for the characterization of the cells with mass cytometry. Purity was additionally confirmed via flow cytometry (NovoCyte Quanteon™). Cultures were maintained at 37°C and 5% CO_2_ throughout the entire protocol.

### Immunofluorescence staining

Immunostaining of hiPSC-neurons was performed as previously described (25). In brief, cells were fixed with 4% paraformaldehyde for 20 min at 4°C and rinsed with PBS. Following a permeabilization step using 0.1% (v/v) Triton X-100 (Sigma) for 30min, the cells were blocked with TBS supplemented with 20% (v/v) serum of the secondary antibody host species for 1h at room temperature. Cells were incubated (4°C) overnight with the primary antibodies diluted in 10% (m/v) milk solution (Sigma) in TBS. After a washing step and a 1h incubation with the secondary antibodies in milk solution on a shaker, cells were again washed and counterstained with DAPI (1 μg/ml, Sigma) for 10 min at 4°C. After a final washing step with distilled water, the sample was mounted using ProLong^®^ Gold antifade reagent (ThermoFisher). The following antibody combinations were used: mouse anti-β-III-tubulin (Tuj1) (2 µg/ml; R&D systems, MAB1195) and rabbit anti-GFAP (1 µg/ml; Abcam, ab7260) in combination with the respective secondary antibodies, goat anti-mouse AF555 (2 µg/ml; Invitrogen, A21425) and goat anti-rabbit FITC (7.5 µg/ml; Jackson ImmunoResearch, 111-096-045), guinea pig anti-NeuN (5 µg/ml; Sigma, ABN90P) in combination with donkey anti-guinea pig Cy3 (7.5 µg/ml; Jackson ImmunoResearch, 706-165-148), and chicken anti-peripherin (1:2000; Invitrogen, PA1-1-10012) in combination with goat anti-rabbit FITC (7.5 µg/ml; Jackson ImmunoResearch, 111-096-045). Immunofluorescent microscopic images of hiPSC-neurons were obtained using a PerkinElmer UltraVIEW Vox spinning disk confocal system.

### Mass cytometry cell staining, acquisition, data pre-processing, and analysis

Unselected hiPSC-macrophages were resuspended in Dulbecco’s Buffered Saline (DPBS, Life Technologies), counted and 2 x 10^6^ cells were aliquoted to staining tubes. First, dead cells were stained with Cell-ID™ Cisplatin (5 μM, CisPt, Standard BioTools) for 5 min at RT and quenched with Maxpar^®^ Cell Staining Buffer (CST, Standard BioTools) supplemented with ethylenediaminetetraacetic acid (2 mM, EDTA, Life Technologies). Next, Fc receptor block (5 µg/ml, Human BD Fc block, BD biosciences) was performed for 10 min at RT, and antibody cocktail of metal-conjugated antibodies (Standard BioTools) (**Table 1**) was added to the cells for surface staining, 30 min at 4°C. Then cells were fixed in freshly prepared formaldehyde solution (1.6%, Life Technologies) for 10 min at RT. Afterward, cells were incubated with Cell-ID Intercalator-Ir (0.125 μM, Ir, Standard BioTools) in Maxpar^®^ Fix and Perm buffer (Standard BioTools) overnight at 4°C. The next day samples were transferred to -80°C and stored until the acquisition. For mass cytometry analysis, samples were shipped on dry ice to the core facility in Genyo (Granada, Spain), thawed, and washed with Maxpar^®^ Cell Acquisition Solution (CAS, Standard BioTools). Cells were acquired on a mass cytometer (HELIOS, Standard BioTools) at an event rate of 400 cells/s together with EQ Four Element Calibration Beads (Standard BioTools). Raw data were normalized using CytoQP function bead_normalize (26). FlowJo software v10.0 was used to analyse normalized data by manual gating. Pre-gating was performed as shown in **Figure S1**.

**Table 1:** Metal-conjugated antibodies and markers used for phenotypical characterization of hiPSC-macrophages by mass cytometry.

Target	Label	Clone	Isotype	Dilution	Cat#
**CD45**	89Y	HI30	Mouse IgG1	1/33,33	3089003
**CD33**	158Gd	WM53	Mouse IgG1	1/33,33	3158001
**CD14**	148Nd	RMO52	Mouse IgG2a	1/33,33	3148010
**CD16**	209Bi	3G8	Mouse IgG1	1/33,33	3209002
**HLA-DR**	143Nd	L243	Mouse IgG2a	1/33,33	3143013
**CD11b (Mac-1)**	144Nd	ICRF44	Mouse IgG1	1/33,33	3144001
**CD64**	146Nd	10.1	Mouse IgG1	1/33,33	3146006
**Ir**	191Ir (DNA1) 193Ir (DNA2)	-	-	-	201192A
**CisPt**	195Pt	-	-	-	201064
**Bead**	165Ho	-	-	-	201078

### Cytokine secretion by hiPSC-macrophages

CD14^+^ hiPSC-macrophages were transfected with poly(dA:dT) (pdAT, 0.25µg/ml; Invivogen) using Lipofectamine 3000 Reagent (Invitrogen) or were treated with Lipofectamine 3000 Reagent alone, according to the manufacturer’s instructions. Unstimulated hiPSC-macrophages were used as a control. After 24h of incubation, supernatants were collected and a LEGENDplex (BioLegend) assay was performed according to manufacturer’s instructions to determine the level of TNF-α, IFN-λ1, IFN-α2, IFN-β and IL-6 as part of the Human Anti-Virus Response Panel. Data were acquired using the NovoCyte Quanteon™ flow cytometer and subsequently analyzed by means of the accompanying LEGENDplex data analysis software (BioLegend) according to manufacturer’s instructions.

### VZV propagation

This study makes use of the recombinant VZV-eGFP/ORF23 strain (27), propagated in the human retinal pigment epithelial cell line ARPE-19 (ATCC, CRL-2303), as described in our earlier work (9). VZV-eGFP/ORF23-infected ARPE-19 cells were cultured in DMEM/F12 (Gibco) supplemented with 10% iFBS (Gibco) and 1% penicillin-streptomycin (Gibco). Titers were determined by an infection foci assay, as described in the protocol of Sloutskin et al. (28) with minor modifications (9). In brief, serial dilutions of VZV-eGFP/ORF23-infected ARPE-19 cells were added to 90% confluent uninfected ARPE-19 cells. Subsequently, fluorescent VZV-eGFP/ORF23 foci were counted were counted after 3 and 7 days for the titre determination using brightfield and eGFP images using an inverted fluorescence microscope (Axio Observer.Z1 with COLIBRI.2 controller, Zeiss).

### Preparation of cell-free VZV

Cell-free (CF) VZV was obtained following lysing VZV-eGFP/ORF23-infected ARPE-19 cells, according to the protocol of Sloutskin et al. (29) with minor modifications as described in our earlier work (9). In brief, ARPE-19 monolayers were infected 1:3 with ARPE-19-associated VZV-eGFP/ORF23 and were harvested at 3 dpi or 4 dpi in ice-cold PBS–sucrose–glutamate–serum buffer (8% 10× PBS, 4% (*m/v*) sucrose, 0.08% (*m/v*) glutamate, 8% iFBS) by scraping the cells off the culture flasks. The cells underwent two cycles of freezing in liquid nitrogen and thawing in a 37°C water bath, followed by sonication for 15 s at 20% amplitude (Vibra-cell™, Sonics & Materials inc.). The resulting VZV cell lysates (further named as CF VZV-eGFP/ORF23) were pooled and stored at -80°C. The absence of intact cells was confirmed by culturing the VZV lysate in DMEM/F12 + 10% iFBS (Gibco™) for seven days. Titers of CF VZV-eGFP/ORF23 were determined by an infection foci assay, as described above.

### Phagocytic properties of hiPSC-macrophages

First, the phagocytic capacity of CD14+ hiPSC-macrophages was assessed using pHrodo Green *S. aureus* Bioparticles™ (Invitrogen) according to the manufacturer’s instructions. In brief, pHrodo bioparticles™ were added to hiPSC-macrophages at a final concentration of 0.1 mg/ml and incubated for 2h at 37°C. hiPSC-macrophages were subsequently harvested and washed with PBS pH 7.4. A live/dead staining was performed by incubating the samples with 7-AAD (1/100; BioLegend) for 10 min at RT, prior to their acquisition by flow cytometry (NovoCyte Quanteon™). Cells-only and beads-only samples served as negative controls. Second, the ability of CD14+ hiPSC-macrophages to phagocytose VZV was assessed by inoculation of the cultures with CF VZV-eGFP/ORF23. More specifically, hiPSC-macrophages were inoculated with CF VZV-eGFP/ORF23 at a multiplicity of infection (MOI) of 0.00149. Similarly, control lysate [CF control (ARPE-19)], derived from non-infected ARPE-19 cells according to the same procedure, was added to hiPSC-macrophages. At 2 and 48 hours post-inoculation, hiPSC-macrophages were harvested and washed with wash buffer (Sheath (BD), 0.1% BSA, 0.05% NaN_3_). A live/dead staining was performed by incubating the samples with 7-AAD (1/100; BioLegend) for 10 min at RT, prior to their acquisition by flow cytometry (NovoCyte Quanteon™). FlowJo software v10.0 was used for subsequent data analysis.

### Stimulation of hiPSC-neurons with pdAT, CF VZV-eGFP/ORF23, and IFN-α2

22-day old hiPSC-neurons were inoculated with CF VZV-eGFP/ORF23 (MOI = 0.00026) or CF control (ARPE-19) for 72h and further cultured for 4 days. For IFN-α2 treatment, cultures were pre-treated with IFN-α2 (2 x 10^5^ U/ml, STEMCELL Technologies) for 24h (at day 21) before inoculation and were treated for the entire duration of the experiment for seven days. Furthermore, 28-day old hiPSC-neurons were transfected with pdAT (2 µg/ml; Invivogen) using Lipofectamine 3000 Reagent (Invitrogen) or were treated with Lipofectamine 3000 Reagent alone for 24h. Unstimulated hiPSC-neurons were used as control. For all different stimulations, hiPSC-neurons were harvested at day 29 and stored in DNA/RNA shield (Zymo Research) at -80°C for downstream RNA isolation and subsequent RT-qPCR and RNA sequencing analyses.

### Stimulation of hiPSC-macrophages with pdAT, CF VZV-eGFP/ORF23, and IFN-α2

CD14+ hiPSC-macrophages were stimulated with CF VZV-eGFP/ORF23 (MOI = 0.00033 or 0.00021) or CF control (ARPE-19), with or without IFN-α2 (1.2 x 10^5^ U/ml, STEMCELL Technologies) treatment for 48h. Furthermore, CD14+ hiPSC-macrophages were transfected with pdAT (0.25 µg/ml; Invivogen) using Lipofectamine 3000 Reagent (Invitrogen) or were treated with Lipofectamine 3000 Reagent alone for 24h. Unstimulated hiPSC-macrophages were used as control. At the end of the 48 or 24h incubation period, respectively, cells were harvested and stored in DNA/RNA shield (Zymo Research) at -80°C for downstream RNA isolation and subsequent RT-qPCR and RNA sequencing analyses.

### RNA extraction and cDNA synthesis

RNA extraction and cDNA synthesis of samples stored in DNA/RNA shield were performed as described previously (9). In brief, samples were thawed at RT and resuspended in an equal volume of lysis buffer (Zymo Research) before starting the Quick-DNA/RNA miniprep (Zymo Research) protocol following the manufacturer’s instructions. After a TURBO™ DNase (Thermo Fisher Scientific) treatment step, purity of RNA samples was checked using a NanoDrop™ 2000 spectrophotometer (Thermo Scientific) and cDNA synthesis was carried out using the SuperScript™ IV First-Strand Synthesis System (Invitrogen™) with oligo(dT)_20_ primers (Invitrogen™).

### RT-qPCR

RT-qPCR was performed as previously described (9). In brief, a mastermix was prepared consisting of 2x SensiFAST TM Probe No-ROX kit (Bioline), 0.1 mg/ml BSA (Thermo Scientific) in PCR-grade water. For RT-qPCR to detect VZV ORF23 and ORF62 transcripts, 500 nM of ORF23, ORF62 and hGAPDH (housekeeping gene) forward (F) and reverse (R) primers, and 100 nM ORF23, 250 nM ORF62 and 250 nM hGAPDH probes were added (**Table 2**). For RT-qPCR to detect OAS1 transcripts, 1 µl of the OAS1 TaqMan TM assay (FAM-MGB, Assay_ID: Hs00973635_m1) and 500 nM hGAPDH forward and reverse primer, and 250 nM hGAPDH probe were added to the mastermix. RT-qPCR reactions were run on the LightCycler^®^ 480 System (Roche Diagnostics, Basel, Switzerland), using following thermal cycling conditions: 1 cycle of 2 min at 95°C (hot start, polymerase activation), followed by 40 cycles of 5 s at 95°C (denaturation) and 20 s at 61°C (annealing and extension). No reverse transcriptase controls (NRT) were run for each sample. Relative quantification to hGAPDH was performed using the Pfaffl Method (32) and represented as fold change or log2 fold change in the graphs.

**Table 2:** Primer and probe sequences used for RT-qPCR.

Gene	Primer/probe sequence	reference
**Human GAPDH_F**	5’-CACATGGCCTCCAAGGAGTAA-3’	(30)
**Human GAPDH_R**	5’-TGAGGGTCTCTCTCTTCCTCTTGT-3’	
**Human GAPDH_Probe**	5’-(Cy5)-CTGGACCACCAGCCCCAGCAAG-(IAbRQSp)-3’	
**VZV ORF23_F**	5’-CTTCTGGACAACAACCGCAA-3’	(9)
**VZV ORF23_R**	5’-CAGATTGTCCCGTGTGTGAC-3’	
**VZV ORF23_Probe**	5’-(TexRed-XN)-ACTGTCCAGCCAACAACCGG-(IabRQSp)-3’	
**VZV ORF62_F**	5’-CCTTGGAAACCACATGATCGT-3’	(31)
**VZV ORF62_R**	5’-AGCAGAAGCCTCCTCGACAA-3’	
**VZV ORF62_Probe**	5’-(HEX)-TGCAACCCGGGCGTCCG-(ZEN/IabRQSp)-3’	

### Monitoring VZV-spreading in hiPSC-neuron/hiPSC-macrophage co-cultures

To evaluate the influence of hiPSC-macrophages on VZV-spreading in neuronal cultures, we employed a XonaChips™ (Xona Microfluidics™) compartmentalized neuronal model separating axons from neuronal cell soma to allow axonal VZV infection, as established previously (9). In brief, hiPSC-neurons were differentiated on one side of the XonaChips™ and were subjected to a 10-fold concentration spatial gradient of rhBDNF and rhGDNF to attract the axons through the microgroove barrier (10 µm width, 150 µm length). A partial medium change was performed every other day. At day 21-22 of neuronal differentiation, 5 x 10^4^ CD14+ hiPSC-macrophages (resuspended in neuronal differentiation medium) were added to the neurons in the somal compartment for 3 days, prior to the inoculation of the neuronal axons with 1.5 x 10^4^ PFU ARPE-19-associated VZV-eGFP/ORF23. At 72 hpi, the medium was changed. Brightfield and immunofluorescent microscopic images were taken at 3 and 7 dpi. For imaging hiPSC-neurons (with hiPSC-macrophages) in XonaChips™, images of complete axonal and somal compartments were obtained by the stitching of multiple 20x images in brightfield and eGFP, acquired by an inverted fluorescence microscope equipped with a motorized stage (Axio Observer.Z1 with COLIBRI.2 controller, Zeiss) and ZEN blue software (Zeiss). Fiji image analysis freeware was used for image processing and analysis (http://fiji.sc). For the quantification of VZV-eGFP/ORF23 foci in the neuronal cultures, images were cut into two, dividing the somal and axonal compartments. By means of manual intensity thresholding, the number of VZV-eGFP/ORF23 foci could be retrieved, as previously described (9).

### RNA-sequencing and analysis

RNA quality was verified using the TapestationTM (Agilent, RNA screentape analysis). RNA sequencing was performed as described in Bartholomeus et al. (33). In brief, RNA samples were prepared with the QuantSeq 30 mRNA-Seq Library Prep Kit FWD for Illumina (Lexogen GmbH) following the standard protocol for long fragments. Obtained cDNA libraries were equimolarly pooled, up to 40 samples for one NextSeq 500 sequencing run (high output v2 kit, 150 cycles, single read, Illumina). Sequencing reads from fastq files were checked for quality using FastQC, trimmed for polyA-tails and further read processing using Trimmomatic v0.36. The reads were then aligned against the human reference genome (GRCh38 from Ensembl) using HISAT2 v2.0.4, and the read count table was assembled using HTSeq. Subsequent expression analysis was performed using the DESeq2 v1.32.0 R package. Differential gene expression (DEG) due to remaining lysate RNA was corrected through stringent adjustment of the comparative p-values based on the source lysate DEGs. This step ensures that the found significant DEGs are due to differences in the macrophage or neural expression, and not due to the addition of the cell lysate. Principal component analysis (PCA) and heatmap plots were assembled using the ggplot2 and pheatmap R packages. For the PCA of the hiPSC-macrophages, data of primary and iPSC-derived myeloid subtypes available from a public dataset were used (34). For the heatmap analysis, the gene expression of the different conditions of hiPSC-macrophages and hiPSC-neurons relative to their unstimulated counterparts, was determined for the genes implicated in different host response pathways, retrieved from the Host Response Panel of Nanostring (https://nanostring.com/products/ncounter-assays-panels/immunology/host-response/). Further pathway and gene set enrichment analysis was performed using the topGO and clusterProfiler R packages.

### Viral transcript analysis

To evaluate whether VZV viral transcripts were captured within the RNA-sequencing the VIRTUS pipeline was used (35). First, VIRTUS.SE.cwl was used to detect viral transcripts by mapping unmapped reads that did not map to the human reference to a VIRTUS defined library of virus references (including the VZV reference fasta). Using a cut-off at a minimum of 100 reads mapped to the VZV reference for the virus to be considered in the sample. A heatmap, using gplots v3.1.3 R package, was generated of raw read counts per sample, this was followed with Wilcoxon tests performed on selected hiPSC-neuronal conditions: unstimulated, control (ARPE 19), VZV-eGFP/ORF23 and VZV-eGFP/ORF23 + IFN-α2. After establishing the presence of VZV viral transcripts in the hiPSC-neurons of the VZV-eGFP/ORF23 and the VZV-eGFP/ORF23 + IFN-α2 conditions. Gene expression differences were analyzed with VIRTUS.SE.singlevirus.cwl, VIRTUS.SE.singlevirus.cwl requires a VZV reference fasta and a VZV gene annotation (36). VIRTUS results were subsequently analysed with Deseq2 v1.38.3 R package and a heatmap was made from the log2(n+1) normalized counts.

### Data representation and statistical analyses

Graphs representing quantitative data were obtained using GraphPad Prism v.8.2.1 software. Statistical analyses were carried out using the statistical software JMP^®^ Pro Version 16. A p-value < 0.05 was considered statistically significant. All data were modelled using a linear mixed-effects model, accounting for the repeated measures, i.e. independent experiments and/or repeated measurements for each observation. The conditions on normality of residuals and homoscedasticity for applying linear mixed-effects models were primarily checked to be in an acceptable range to perform this type of analysis. For RT-qPCR data of OAS1 mRNA expression by hiPSC-macrophages and for the quantification of VZV-eGFP/ORF23 foci in the axonal compartment, log-transformed data were used. For the quantification of VZV-eGFP/ORF23 foci in the somal compartment (x), log(x+1)-transformation was used, considering zero values in the original dataset. *Post-hoc* analyses for linear mixed-effects models were carried out with Tukey HSD correction for multiple comparisons.

## Results

### Phenotype and immune responsiveness of hiPSC-neurons

**Figure 1:**
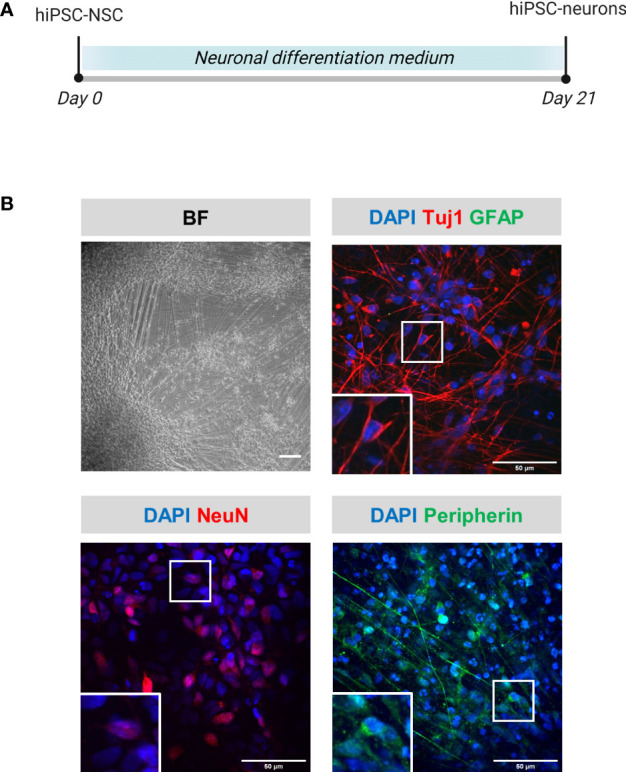
Phenotypical characterization of hiPSC-neurons. **(A)** Schematic overview of the differentiation protocol of hiPSC-neurons. **(B)** Representative brightfield (BF) and immunofluorescent microscopic images of hiPSC-neurons obtained at the end of the differentiation protocol (day 21). hiPSC-neurons were stained for the neuronal markers Tuj1 and NeuN, the astrocyte marker GFAP and the PNS neuronal marker peripherin. Scale bars indicate 100µm and 50µm, for BF and immunofluorescent images, resp. (hiPSC-NSC, hiPSC-derived neural stem cells).

**Figure 2:**
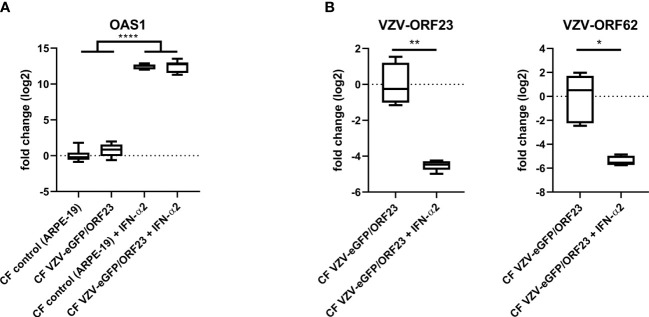
hiPSC-neurons are irresponsive to VZV-infection without exogenous IFN-α2. **(A)** OAS1 RT-qPCR data of hiPSC-neurons after infection with CF VZV-eGFP/ORF23 or CF control (ARPE-19), with or without IFN-α2 treatment. For each condition n = 5-6, measured in duplicate. **(B)** VZV-ORF23 and VZV-ORF62 RT-qPCR data of hiPSC-neurons after infection with CF VZV-eGFP/ORF23 or CF control (ARPE-19), with or without IFN-α2 treatment. For each condition n = 5-6, measured in duplicate. *p<0.05, **p<0.01, ****p<0.0001.

hiPSC-neurons were generated from a previously established hiPSC-derived neural stem cell line (hiPSC-NSC) (25), whereby its differentiated progeny adopts a peripheral nervous system (PNS)-like neuronal phenotype after 3 weeks of culture (**Figure 1A**), as demonstrated by immunocytochemical analyses for NeuN, Tuj1 and Peripherin expression (**Figure 1B**). Obtained hiPSC-neuron cultures do not contain contaminating GFAP+ astrocytes (**Figure 1B**). In a preceding study (9), we already demonstrated that this hiPSC-neuron population was highly susceptible for productive VZV infection with a cell-free (CF) preparation of the reporter gene modified APRE19-associated VZV-eGFP/ORF23 strain. However, despite an ongoing productive VZV infection, no upregulation of mRNAs for IFN-α2 or Interferon-Stimulated Genes (ISGs) encoding anti-viral response proteins (PKR, ISG15, Mx1 and OAS1) was detected in hiPSC-neurons (9). To recapitulate this feature, the hiPSC-neuron cultures prepared for use in this study also did not show upregulation of OAS1 mRNA upon infection with CF VZV-eGFP/ORF23 as compared to CF control (ARPE-19) lysate (**Figure 2A**). As positive control for immune responsiveness of hiPSC-neurons, OAS1 mRNA was significantly upregulated in non-infected (CF control (ARPE-19)) and CF VZV-eGFP/ORF23-infected hiPSC-neurons following stimulation with IFN-α2 (**Figure 2A**). Likewise, both VZV ORF23 and VZV ORF62 mRNA was significantly reduced in CF VZV-eGFP/ORF23 infected hiPSC-neurons treated with IFN-α2 as compared to non-treated CF VZV-eGFP/ORF23 infected hiPSC-neuron cultures (**Figure 2B**). These results summarize our previous findings whereby we hypothesised that although hiPSC-neurons do not activate an intrinsic antiviral immune response upon VZV infection, they are sensitive to environmental stimuli (e.g. IFN-α2) to suppress a productive VZV infection (9).

### Phenotypic and molecular characterisation of hiPSC-macrophages

**Figure 3:**
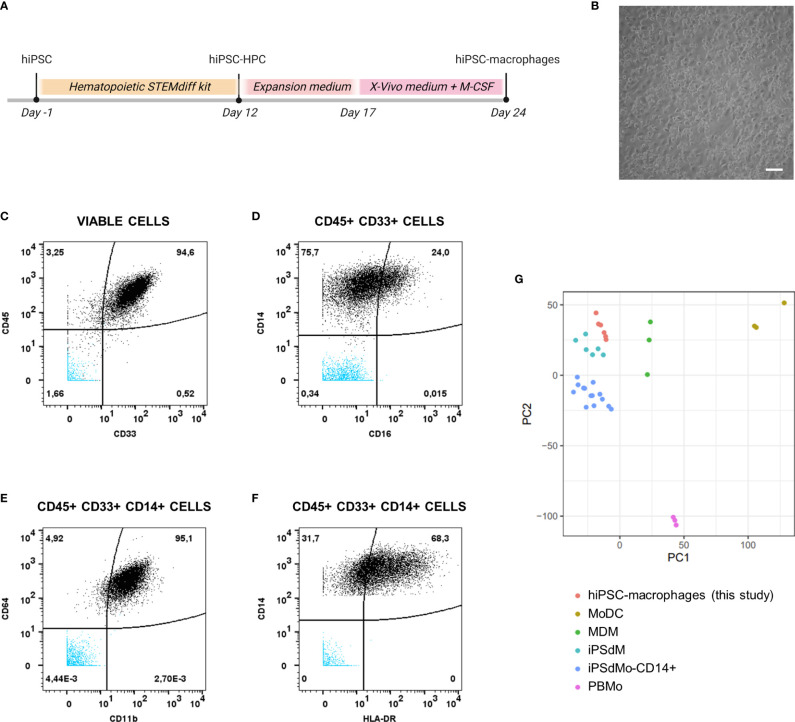
Phenotypical and molecular characterization of hiPSC-macrophages. **(A)** Schematic overview of the differentiation protocol of hiPSC-derived macrophages. **(B)** Representative brightfield microscopic image of hiPSC-derived macrophages obtained at the end of the differentiation protocol (day 24). Scale bar 50µm. **(C–F)** Mass cytometry analysis of hiPSC-derived macrophages immunolabeled for the hematopoietic cell marker CD45, the myeloid marker CD33 and the monocyte/macrophage markers CD14, CD16, CD64, CD11b, and HLA-DR. Data shown here are from a representative sample, i.e. sample from the experiment of which the proportion of marker positive cells is closest to the overall mean value of the three independent experiments combined, depicted in **Table 3**. The population subsets are given on top of the corresponding plots. Unstained hiPSC-macrophage population is visible in blue. **(G)** Principal component analysis of expressed genes obtained by bulk RNA-sequencing for the generated hiPSC-macrophages derived from two independent differentiations (n=3 per differentiation) along with primary and iPSC-derived myeloid subtypes available from a public dataset (34). The top 2 principal components were used. (hiPSC-HPC, hiPSC-derived hematopoietic progenitor cell; MoDC, human primary monocyte-derived dendritic cell; MDM, human primary monocyte-derived macrophages; iPSdM, iPSC-derived macrophages; iPsdMo-CD14+, iPSC-derived CD14+ monocytes; PBMo, human primary monocytes).

hiPSC-macrophages were generated from a previously established hiPSC line (25), the same that was used to generate the above described hiPSC-NSC line. Following hiPSC differentiation into hematopoietic progenitor cells (hiPSC-HPC) using a commercially available differentiation kit, a sequential expansion, and macrophage differentiation step (**Figure 3A**) was applied resulting in a population of round-shaped macrophage-like cells (**Figure 3B**). This population was then further characterised for specific monocyte/macrophage membrane marker expression using mass cytometry analysis (**Figures 3C–F** and **Table 3**). The majority of cells analysed were identified as hematopoietic cells of the myeloid lineage, as determined by CD45 and CD33 marker positivity, respectively (**Figure 3C**). Within the CD45+CD33+ population, virtually all cells express the monocyte/macrophage marker CD14, with variable co-expression of FcγRIII (CD16) (**Figure 3D**). Within the CD45+CD33+CD14+ cell population, high expression of ITGAM (CD11b) and FcγRI (CD64) is noted (**Figure 3E**), while HLA-DR displays variable expression at base-line conditions (**Figure 3F**). This macrophage-like phenotype was further confirmed using bulk RNA-Seq analysis (**Figure 3G**), comparing the gene expression profile of our hiPSC-macrophages with those of different myeloid cell populations derived from a publicly available bulk RNA-sequencing dataset (34). Principal component analysis (PCA) using the first two PCs shows clustering of our hiPSC-macrophages with iPSC-derived CD14+ monocytes (iPsdMo-CD14+), human primary monocyte-derived macrophages (MDM) and iPSC-derived macrophages (iPSdM). By contrast, our hiPSC-macrophages display less similarity to human primary monocytes (PBMo) and human primary monocyte-derived dendritic cells (MoDC). These data provide evidence that the applied hiPSC-macrophage differentiation protocol results in the generation of an iPSC-derived macrophage-like cell population similar to those described by others and closely resembling their native *in vitro* counterpart (34).

**Table 3:** Quantitative mass cytometry data.

	Mean	SD
**Viable cells**	95.5	0.9
CD45+/CD33+	85.5	14.1
CD14+	99.7	0.1
CD16+	15.2	8.5
CD11b+	84.6	11.7
CD64+	99.8	0.3
HLA-DR+	63.7	35.9

hiPSC-macrophages immunolabeled for the hematopoietic cell marker CD45, the myeloid marker CD33 and the monocyte/macrophage markers CD14, CD16, CD64, CD11b, and HLA-DR. For each marker the mean proportion of cells from three independent experiments is given, together with the standard deviation (SD).

### Functional characterisation of hiPSC-macrophages

**Figure 4:**
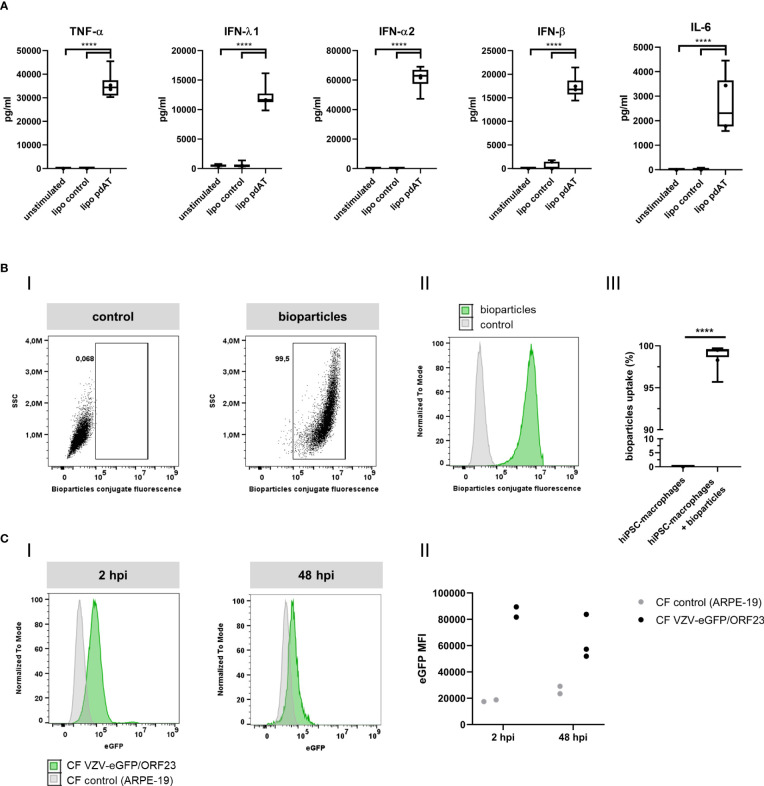
Functional characterization of hiPSC-macrophages. **(A)** Anti-viral cytokine concentrations in the culture supernatants of hiPSC-macrophages transfected with 0.25 µg/ml pdAT using lipofectamine, stimulated with lipofectamine alone or unstimulated for 24h, obtained by LEGENDplex. Data were obtained from two independent experiments with n = 5-6 per condition, measured in duplicate. The box plots indicate median and interquartile range, whiskers indicate the minimum and maximum values of the two independent experiments combined. The dots indicate the median value of each individual experiment. **(B)** Phagocytosis of fluorescent *S. aureus* bioparticles by hiPSC-macrophages. (i) Representative image of gating strategy to determine the relative number of hiPSC-macrophages that phagocytosed fluorescent *S. aureus* bioparticles. (ii) Representative histogram (i.e. histogram from sample with median bioparticle uptake closest to the overall median bioparticle uptake of all experiments combined from Figure iii) of hiPSC-macrophages demonstrating their phagocytic capacity. Grey, control hiPSC-macrophages; green, hiPSC-macrophages with addition of bioparticles. (iii) Quantification of the relative number of phagocytic hiPSC-macrophages, represented as percentage bioparticles uptake in the graph. Data obtained from two independent experiments with n = 1-2 for control hiPSC-macrophages (hiPSC-macrophages) and n = 3-6 for hiPSC-macrophages with addition of bioparticles (hiPSC-macrophages + bioparticles) per experiment. The box plots indicate median and interquartile range, whiskers indicate the minimum and maximum values of the two independent experiments combined. The dots indicate the median value of each individual experiment. **(C)** Phagocytosis of fluorescent CF VZV-eGFP/ORF23 by hiPSC-macrophages at 2hpi and 48hpi. (i) Representative histogram of hiPSC-macrophages demonstrating their phagocytic capacity of CF VZV-eGFP/ORF23 at 2hpi and 48hpi. Grey, hiPSC-macrophages incubated with CF control (ARPE-19); green, hiPSC-macrophages incubated with CF VZV-eGFP/ORF23. (ii) Median fluorescence intensity (MFI) of eGFP signal of hiPSC-macrophages incubated with CF control (ARPE-19) or CF VZV-eGFP/ORF23 for 2 or 48h. The dots indicate values of individual experiments. (pdAT, poly(dA:dT); hpi, hours post-infection) ****p<0.0001.

**Figure 5:**
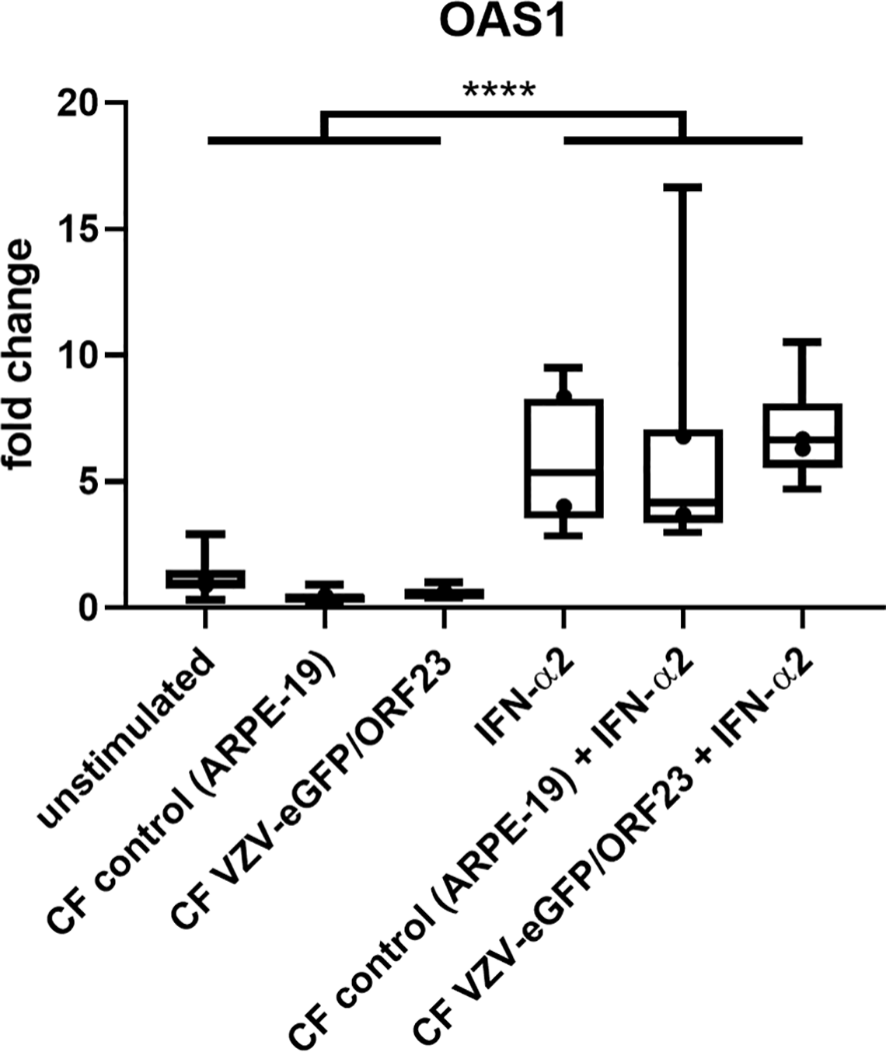
hiPSC-macrophages do not upregulate OAS1 mRNA following VZV-infection without exogenous IFN-α2. OAS1 RT-qPCR data of hiPSC-macrophages stimulated with CF VZV-eGFP/ORF23 or CF control (ARPE-19), with or without simultaneous IFN-α2 treatment for 48h. Only IFN-α2-stimulated and unstimulated hiPSC-macrophages were included in this experimental set-up. Data were obtained from two independent experiments with per experiment n = 4-6 per condition, measured in duplicate. The box plots indicate median and interquartile range, whiskers indicate the minimum and maximum values of the two independent experiments combined. The dots indicate the median value of each individual experiment. ****p<0.0001.

To demonstrate innate immune responsiveness of the cultured hiPSC-macrophages, we first lipofected hiPSC-macrophages with pdAT, a well-known synthetic DNA analogue that activates the type I and III IFN-signalling pathway. This resulted in significant production of high levels of anti-viral cytokines, including TNF-α, IFN-λ1, IFN-α2, IFN-β and IL-6, by hiPSC-macrophages (**Figure 4A**). Next, we investigated whether hiPSC-macrophages were able to perform phagocytosis of pHrodo Green *S. aureus* Bioparticles. Within 2 hours, more than 95% of hiPSC-macrophages efficiently phagocytosed pHrodo Green *S. aureus* Bioparticles as measured by flow cytometric analysis (**Figure 4B**). Finally, and specifically within the context of the main research question of this manuscript, we also assessed whether hiPSC-macrophages were able to actively phagocytose CF VZV-eGFP/ORF23. Flow cytometric analysis demonstrated that a clear eGFP signal could be detected within hiPSC-macrophages already after 2 hours following exposure with CF VZV-eGFP/ORF23, which - although diminished - could still be detected at 48 hours post-challenge (**Figure 4C**). Surprisingly, even though CF VZV-eGFP/ORF23 was efficiently phagocytosed by hiPSC-macrophages, no upregulation of OAS1 mRNA was detected as compared to unstimulated or CF control (ARPE-19) stimulated hiPSC-macrophages (**Figure 5**). Here again, stimulation with IFN-α2 served as positive control to demonstrate the intrinsic capacity of hiPSC-macrophages to mount an antiviral response, as shown by significant upregulation of OAS1 mRNA following IFN-α2 treatment, even under the condition of CF VZV-eGFP/ORF23 infection. Concluding, the above-described experiments demonstrate the phagocytic capacity (pHrodo Green *S. aureus* Bioparticles) as well as the immune responsiveness (pdAT stimulation) of our cultured hiPSC-macrophages, but at the same time suggests the high immune-evasive properties of VZV and/or the low innate immune reactivity of macrophages towards VZV.

### hiPSC-macrophages are unable to control VZV-infection in hiPSC-neurons in a compartmentalized neuronal model

**Figure 6:**
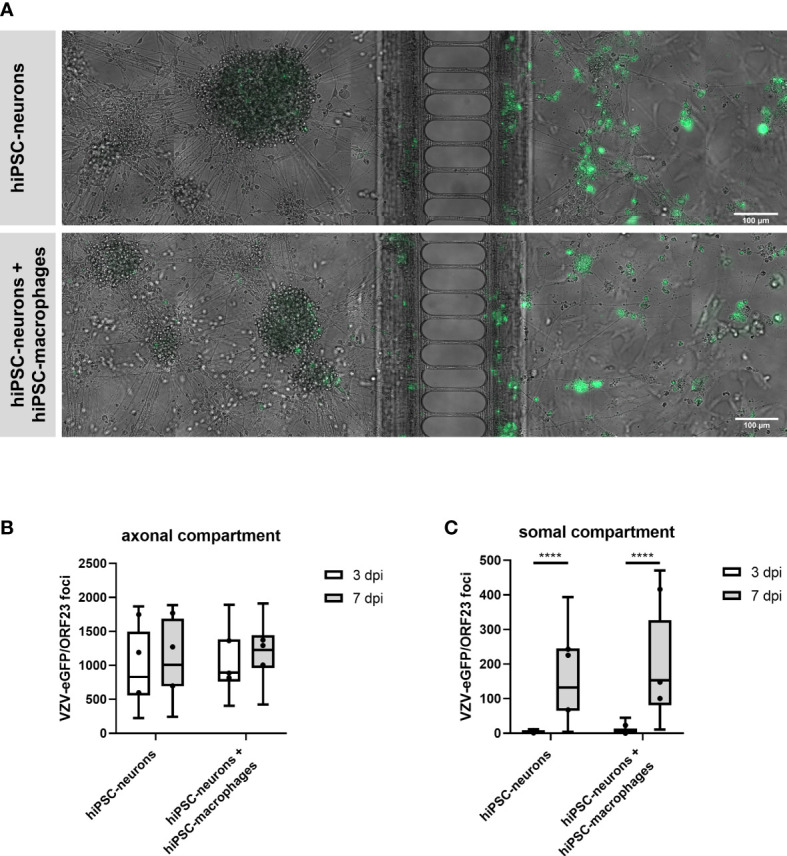
hiPSC-macrophages are unable to control VZV-infection in hiPSC-neurons in a compartmentalized neuronal model. **(A)** Representative images of hiPSC-neuronal cultures with or without hiPSC-macrophages in the somal compartment at day 7 after inoculation with ARPE-19-associated VZV-eGFP/ORF23 in the axonal compartment. Scale bar 100 µm. **(B, C)** Quantification of the number of VZV-eGFP/ORF23 foci present in the axonal compartment **(B)** or somal compartment **(C)** at 3 dpi and 7 dpi. Data were obtained from three independent experiments with n = 4-8 for hiPSC-neurons and n = 5-8 for hiPSC-neurons + hiPSC-macrophages per experiment. The box plots indicate median and interquartile range, whiskers indicate the minimum and maximum values of the three independent experiments combined. The dots indicate the median value of each individual experiment. ****p<0.0001.

In this part of our study we investigated whether hiPSC-macrophages, upon co-culture with hiPSC-neurons, were able to prevent a productive VZV infection in hiPSC-neurons in a compartmentalized neuronal model (XonaChips™, **Figure 6A**). In this previously established compartmentalized model (9), interconnected neuronal cell bodies are physically separated in the somal compartment (**Figure 6A**, left side) from a limited number of outgrowing axons in the axonal compartment (**Figure 6A**, right side). Following axonal infection with cell-associated (ARPE-19) VZV-eGFP/ORF23, productive infection of hiPSC-neurons in the somal compartment can easily be monitored by quantifying the number of eGFP (VZV) foci appearing over time. By adding hiPSC-macrophages to the somal compartment, thereby mimicking cellular innate immune recognition of VZV-infected neurons by tissue-patrolling macrophages, we here aimed to investigate whether or not macrophages can act as a stand-alone immune cell population to control productive VZV infection of hiPSC-neurons. Quantification of the number of VZV foci in the axonal compartment, which are derived from VZV-eGFP/ORF23 in ARPE-19 cells, served as internal control (i.e. equal amounts of VZV were provided to all experimental conditions) whereby no significant differences were observed between day 3 to day 7 post-infection within and between the two experimental conditions, namely hiPSC-neurons and hiPSC-neurons + hiPSC-macrophages (**Figure 6B**). Within the somal compartment, a significant increase of VZV-eGFP/ORF23 foci was counted between day 3 and day 7 post-infection for both the hiPSC-neuron condition and the hiPSC-neuron + hiPSC-macrophage condition (**Figure 6C**), thereby demonstrating successful productive VZV infection of hiPSC-neurons in the somal compartment. However, and in contrast to our initial expectations, the presence of hiPSC-macrophages did not reduce the number of VZV-eGFP/ORF23 foci at day 7 post-infection (**Figure 6C**). Based on this experimental outcome, and in agreement with the results described above (**Figure 5**), we may hypothesise that an absent or low innate immune reactivity of macrophages following VZV encounter renders them unable to control a productive neuronal VZV infection without the help of additional immune cell populations and/or the activation of appropriate immune signalling cascades.

### Experimental setup RNA-seq analysis

In order to provide further evidence for the hypothesis suggested above, we performed an extensive bulk RNA-Seq analysis on both hiPSC-macrophages and hiPSC-neurons under various stimulatory conditions. For hiPSC-macrophages, the following conditions were included: (i) unstimulated hiPSC-macrophages (two differentiations, n=3 and n=3), (ii) lipofectamine control for hiPSC-macrophages (two differentiations, n=2 and n=3; 24 hours after treatment), (iii) hiPSC-macrophages after lipofection with pdAT (two differentiations, n=3 and n=3; 24 hours after treatment), (iv) hiPSC-macrophages challenged with CF control (ARPE-19) lysate (two differentiations, n=3 and n=3; 48 hours after challenge) and (v) hiPSC-macrophages challenged with CF VZV-eGFP/ORF23 lysate (two differentiations, n=3 and n=3; 48 hours after VZV challenge). Conditions (i), (ii) and (iii) refer to the experiments presented in **Figure 4A**, i.e. cytokine production by hiPSC-macrophages following pdAT stimulation. Conditions (i), (iv) and (v) refer to experiments presented in **Figure 5**, i.e. lack of OAS1 mRNA upregulation following challenge of hiPSC-macrophages with CF VZV-eGFP/ORF23 lysate. For hiPSC-neurons, the following conditions were included: (i) unstimulated hiPSC-neurons (two differentiations, n=3 and n=3), (ii) lipofectamine control for hiPSC-neurons (two differentiations, n=3 and n=3; 24 hours after treatment), (iii) hiPSC-neurons after lipofection with pdAT (two differentiations, n=3 and n=3; 24 hours after treatment), (iv) hiPSC-neurons challenged with CF control (ARPE-19) lysate (two differentiations, n=3 and n=3; 7 days after challenge), (v) hiPSC-neurons infected with CF VZV-eGFP/ORF23 lysate (two differentiations, n=3 and n=3; 7 days after VZV infection) and (vi) hiPSC-neurons stimulated/infected with CF VZV-eGFP/ORF23 lysate and treated with IFN-α2 (two differentiations, n=2 and n=2; 8 days after IFN-α2 treatment/7 days after VZV infection). Conditions (iv), (v) and (vi) refer to the experiments presented in **Figure 2A**, i.e. lack of OAS1 mRNA upregulation following CF VZV-eGFP/ORF23 infection of hiPSC-neurons and OAS1 mRNA upregulation following IFN-α2 treatment of CF VZV-eGFP/ORF23 infected hiPSC-neurons.

### RNA-seq analysis highlights immune competence of both hiPSC-neurons and hiPSC-macrophages, but absence of a strong host immune response following, respectively, VZV infection or challenge

**Figure 7:**
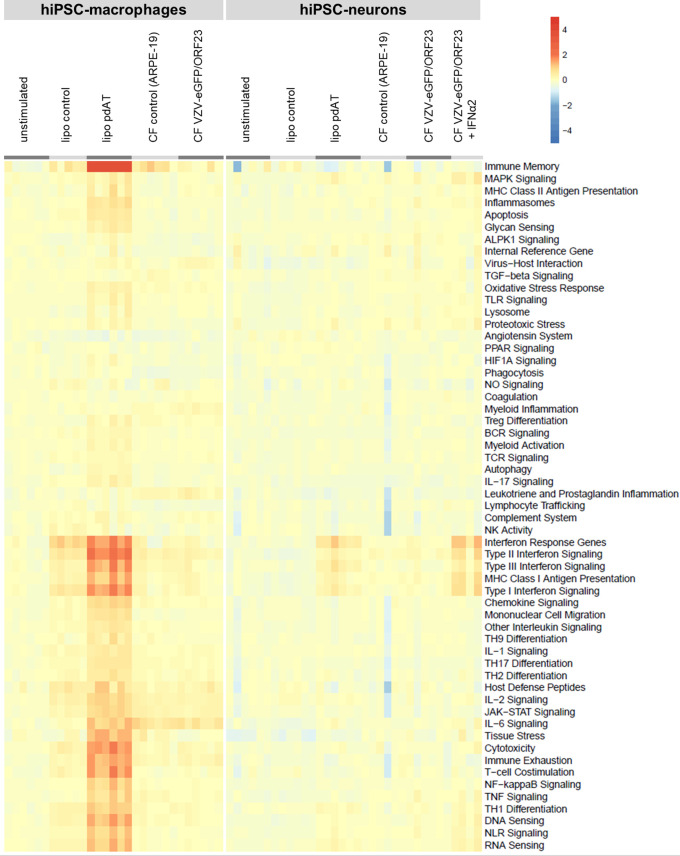
Transcriptomic profile of hiPSC-macrophages and hiPSC-neurons shows absence of a strong host immune response following VZV challenge, despite their immune competence. Heat-map shows the up-or downregulation of genes implicated in different host response pathways (retrieved from Nanostring) for the different conditions relative to the unstimulated control condition for hiPSC-macrophages or hiPSC-neurons.

Given this extensive RNA-Seq dataset, a heat map (**Figure 7**) was generated for a selection of key genes covering main aspects of host response, including host susceptibility, interferon response, innate immune cell activation, adaptive immune response and homeostasis. The list of key genes used for this analysis, including their category annotation, was modified from the gene/annotation list from the NanoString Host Response panel and in modified form provided as supplementary file 1. For both hiPSC-macrophages and hiPSC-neurons, we have plotted up- and down-regulated gene categories under different stimulatory conditions versus the control unstimulated cells. In addition, the level of up- or down-regulation was also taken into account when comparing hiPSC-macrophages and hiPSC-neurons. The resulting heat map clearly illustrates that, while hiPSC-macrophages strongly react upon lipofection with the viral mimic pdAT, their response in terms of activation of host response pathways is nearly absent (or very minimal) upon challenge with CF VZV-eGFP/ORF23. Likewise, hiPSC-neurons, as compared to hiPSC-macrophages, only moderately react upon lipofection with pdAT, while again VZV-eGFP/ORF23 infection of hiPSC-neurons does not lead to activation of host response pathways. However, in agreement with our preceding data [(9) and **Figures 2A, B**] hiPSC-neurons do mount a strong antiviral response upon treatment with IFN-α2.

### Viral transcript analysis

**Figure 8:**
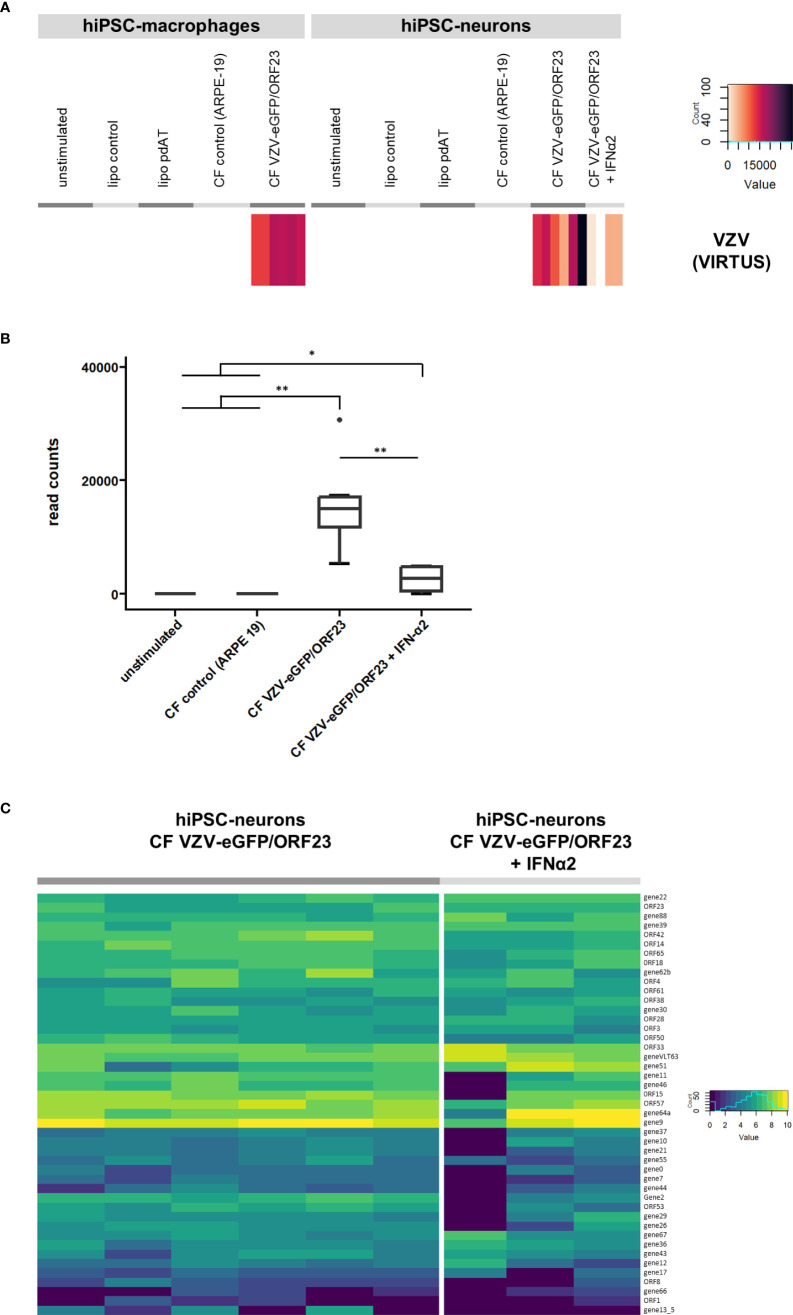
Viral transcript analysis. **(A)** VZV viral transcripts recovered in VZV challenged hiPSC-macrophages and VZV-infected hiPSC-neurons. Heatmap showing the raw read count of reads mapping to the VZV reference genome, for the different conditions of hiPSC-macrophages or hiPSC-neurons. **(B)** Boxplot showing the reduction of VZV transcripts in hiPSC-neurons infected with VZV following treatment with exogenous IFN-α2. **(C)** Gene expression of VZV recovered in VZV-infected hiPSC-neurons. Heatmap showing the log2(n+1) normalized counts of reads mapping to VZV genes, for hiPSC-neurons infected with CF VZV-eGFP/ORF23 and those additionally treated with IFN-α2. *p<0.05. **p<0.01.

Lastly we performed an additional VIRTUS bioinformatics analysis (35) on our RNA-Seq datasets to detect VZV-specific transcripts within bulk RNA-Seq data. As shown in **Figure 8A**, VZV-specific transcripts were, as expected, detected in hiPSC-macrophages challenged with CF VZV-eGFP/ORF23 and in hiPSC-neurons infected with CF VZV-eGFP/ORF23 with and without IFN-α2 treatment. In agreement with our previous (9) and above-described data, IFN-α2 treatment significantly reduces the total number of VZV viral reads in VZV-infected hiPSC-neurons (**Figure 8B**). Further analysis only focused on hiPSC-neurons as the infectious CF VZV-eGFP/ORF23 lysate was removed during cell culture, while hiPSC-macrophages phagocytosed the CF VZV-eGFP/ORF23 lysate (and thus obtained reads may not reflect VZV transcriptome in hiPSC-macrophages). As shown in **Figure 8C**, we demonstrate the presence of a vast majority of VZV-specific transcripts associated with lytic infection in hiPSC-neurons infected with CF VZV-eGFP/ORF23. Furthermore, even though IFN-α2 treatment was able to reduce the total number of VZV transcripts (**Figure 8B**), the VZV-specific transcriptome in IFN-α2 treated hiPSC-neurons infected with CF VZV-eGFP/ORF23 did not display major alterations. Although further investigation may be needed, this suggest that IFN-α2 treatment only suppresses VZV replication, but does not alter its intrinsic transcriptomic profile.

## Discussion

While *in vitro* stem cell-derived neuronal models are of great interest for studying neuron-pathogen interactions (8), these models also hold the potential to study multicellular neuro-immune interactions. Within the context of VZV, we and others have used a compartmentalized neuronal model to achieve productive infection of neuronal cultures following axonal VZV infection (8, 9, 12, 13). While we have shown in our previous study that exogenously administered IFN-α2 can efficiently limit VZV spreading throughout PNS-like hiPSC-derived neuronal cultures, we hypothesised that circulating innate immune cells, such as macrophages, could be a potential source of IFN-α2 production upon VZV infection or contact with VZV-infected neurons. This hypothesis was further supported by our preceding observation that peripheral blood mononuclear cells (PBMC) from several paediatric patients with inborn errors in RNA polymerase III exhibited defective ability to produce IFN-encoding mRNAs, including IFN-α2 mRNA (17). Since our previously established compartmentalised neuronal VZV infection model was initiated from reprogrammed commercially available foreskin fibroblasts, no autologous primary macrophages could be added to our model. Therefore, we have here established hiPSC-derived macrophages from the same iPSC-line as used for generation of the compartmentalized hiPSC-derived neuronal model. Despite profound characterisation (mass cytometry and RNA-Seq, **Figure 3**) and demonstration of their immune competence (phagocytosis of bioparticles and CF VZV-eGFP/ORF23 lysate, cytokine production following pdAT stimulation and responsiveness to IFN-α2, **Figures 4**, **5**), challenge with VZV did not lead to strong innate immune reactivity in hiPSC-macrophages (**Figures 5**, **7**) and subsequent control of VZV spread in hiPSC-neurons (**Figure 6**). The importance of this study is that even though VZV-challenge has a transcriptomic influence on hiPSC-macrophages (see below), these responses are insufficient to allow them as a stand-alone immune cell population to control a productive VZV-infection of hiPSC-neurons.

Regarding innate immune reactivity of monocytes/macrophages towards VZV, current literature provides rather inconclusive answers. At one end, the observations that both the THP-1 monocyte cell line and primary monocytes can produce IL-6, IL-8 and IL-1β on the protein level following VZV infection imply the active recognition of VZV by TLR2 and NLRP3 (22–24, 37). Although not shown in this manuscript, in our hands we did not detect significant levels of IL-1β, IL-6, TNF-α, IP-10, IFN-Λ1, IL-8, IL-12p70, IFN-α2, IFN-Λ2/3, GM-CSF, IFN-β, IL-10 or IFN-γ at the protein level when hiPSC-macrophages were in co-culture with VZV-infected hiPSC-neurons. This is in line with Huch et al. (20) who investigated IFN-α2 secretion by plasmacytoid dendritic cells (pDC) and noted that pDC upon VZV infection not only did not secrete IFN-α2 but even more importantly were completely unable to secrete IFN-α2 upon co-stimulation with the TLR9 agonist ODN2216. Also, from our preceding work we are aware that detected levels of IFN-α2 mRNA in VZV-stimulated PBMC is approximately 2000-fold lower as compared to pdAT-stimulated PBMC, possibly explaining the absence of cytokine secretion on the protein level by our hiPSC-macrophages (17). Nevertheless, we cannot rule out that cytokine secretion was below detectable levels. However, the minute amounts that in this case may have been secreted are most likely insufficient to exert a direct suppressive effect on VZV. This is also reflected by the inability of our hiPSC-macrophages to suppress an ongoing VZV infection in hiPSC-neurons (**Figure 6**). The observation that macrophages cannot act as a stand-alone population to suppress an ongoing VZV infection in hiPSC-neurons may also be further supported by Kennedy et al., who performed a comprehensive characterization of the interaction of VZV with monocytes (4). Although no cytokine secretion was investigated in this study, they observed downregulation of CD14, HLA-DR, CD11b and M-CSF, as well as impaired monocyte to macrophage maturation leading to loss of cell viability. Despite not investigated in detail in our study, we did not observe apparent cell death among VZV-challenged hiPSC-macrophages. This however may be due to the experimental set-ups applied where hiPSC-macrophages encountered VZV either via a CF VZV-eGFP/ORF23 lysate or via contact with VZV-infected hiPSC-neurons. Obviously, we cannot rule out that literature and our data are biased by the use of a cell line and/or limited number of PBMC donors and/or iPSC lines, reflecting heterogeneity and variability in innate immune responsiveness.

Even though hiPSC-macrophages were unable to directly suppress an ongoing VZV-infection in hiPSC-neurons, we do not claim a fully redundant role for macrophages. One important future direction may be to extend the current innate neuron-macrophage model with components of the adaptive immune system, thereby investigating the influence of the CD4+ T-cell interaction with antigen-presenting macrophages, as well as their combined effect on VZV-spread in hiPSC-neurons. However, such tri-partite studies would require the availability of autologous PBMC to obtain VZV-specific CD4+ T-cells. This approach may not be illogical for the further elucidation of neuro-immune interactions in the context of VZV since, for example, cytokine detection following VZV challenge/infection seems to be more successful when applied to PBMC samples (17), where complex interaction between monocytes and T-cells take place, as is at the site of neuronal VZV-infection *in vivo*. Indeed, histological characterisation of active herpes zoster demonstrated the presence of a prominent widespread infiltrate of inflammatory cells, predominantly by small lymphocytes and macrophages (38). With the notion that specific T cell subsets are likely to play an important role in controlling VZV in neurons, it remains to be investigated whether they directly act on neurons or act *via* or depend on antigen-presenting macrophages.

With hiPSC-macrophages being unable to perform a direct suppressing effect on VZV spreading in hiPSC-neurons, as well as the observed lack of cytokine secretion, we performed an extensive RNA-Seq analysis on hiPSC-neurons and hiPSC-macrophages. Fully in line with our previous data (9), hiPSC-neurons were irresponsive to VZV infection in terms of host response genes (**Figure 7**) and upon DEG analysis of the full dataset, no transcripts were found significantly up- or down-regulated (**Table 4**). Previously, Markus et al. also performed a comparative microarray analysis on VZV-infected human embryonic stem cell (hESC)-derived neurons and human fibroblasts (39). In their study, 378 DEGs were identified in hESC-neurons following VZV-infection, with a specific focus on the upregulation of anti-apoptotic gene expression. In contrast, in human fibroblasts 2609 DEGs were identified following VZV infection, with a specific focus on the upregulation of pro-apoptotic gene expression. In our study, we could not detect the upregulation of anti-apoptotic gene expression, which may either be due to differences in infectivity and/or timing of the RNA-Seq analysis, or alternatively the fibroblast origin of our hiPSC-neurons (before reprogramming to iPSC and differentiation into hiPSC-NSC) may have interfered with the upregulation anti-apoptotic gene transcripts. In future research, comparative studies between (multiple) hESC- and hiPSC-derived models are required to solve this issue. Nevertheless, we here demonstrated that hiPSC-neurons were clearly able to activate type I interferon signalling pathways following lipofection with pdAT and following IFN-α2 treatment, thereby demonstrating the presence of a functional innate immune signalling (**Figure 7**; **Table 4** and **Supplementary File 2**). However, as compared to hiPSC-macrophages, the response towards lipofection with pdAT in hiPSC-neurons was less pronounced, both in number of DEGs as well as magnitude of gene transcription. (**Figure 7**; **Table 4** and **Supplementary File 3**). Even though hiPSC-macrophages are fully susceptible to activate type I interferon signalling, VZV challenge did not lead to this type of innate immune activation. Nevertheless, in contrast to hiPSC-neurons, 1007 DEGs were identified following VZV challenge. As can be noted from the Top-5 GO biological processes associated, it is at one end clear that this stimulation is insufficient to exert a direct VZV suppressive effect on hiPSC-neurons, but on the other hand does not exclude the absence of a strong regulatory function for macrophages in controlling neuronal VZV infection.

**Table 4:** DEG analysis and Top-5 GO biological processes.

	Comparison	DEG analysis (Log2)	Top-5 GO biological processes
**hiPSC-neurons**	lipo control lipo pdAT	100 ↑ (0,3 – 6,6) 45 ↓ (-0,3 – -2,4)	1. type I interferon signalling pathway 2. defense response to virus 3. interferon-gamma-mediated signalling pathway 4. negative regulation of viral genome replication 5. antigen processing and presentation of exogenous peptide antigen via MHC class I, TAP-independent
	CF CONTROL (ARPE-19) CF VZV-ORF23/eGFP	none	none
	CF CONTROL (ARPE-19) CF VZV-ORF23/eGFP + IFN-α2	58 ↑ (0,8 – 7,4) 1 ↓ (2,2)	1. type I interferon signalling pathway 2. antigen processing and presentation of exogenous peptide antigen via MHC class I, TAP-independent 3. defense response to virus 4. interferon-gamma-mediated signaling pathway 5. negative regulation of viral genome replication
**hiPSC-macrophages**	lipo control lipo pdAT	2217 ↑ (0,4 – 10,5) 1196 ↓ (-0,4 – -7,4)	1. type I interferon signalling pathway 2. inflammatory response 3. cellular response to lipopolysaccharide 4. humoral immune response 5. interferon-gamma-mediated signalling pathway
	CF CONTROL (ARPE-19) CF VZV-ORF23/eGFP	510 ↑ (0,3 – 2,1) 497 ↓ (-0,3 – -3,6)	1. neutrophil degranulation 2. wound healing 3. negative regulation of T-helper 2 cell differentiation 4. neutrophil chemotaxis 5. positive regulation of angiogenesis

DEG analysis showing the number of up- and down-regulated genes including the Log2 range observed for the different comparisons indicated, including the Top-5 GO biological processes associated with the observed DEGs.

Additionally, we also attempted to investigate the VZV transcriptome in hiPSC-derived neurons infected with CF VZV-eGFP/ORF23, based on our bulk RNA-Seq analysis. Even though this pipeline may need further validation, it is interesting to note the high expression level of ORF9 (gene9, VP22; **Figure 8C**), which was recently demonstrated to be an antagonist of the DNA sensor cGAS (40). Given this high expression, VP22 may have inhibited cGAMP production and DNA‐triggered type I IFN induction upon VZV-infection of hiPSC-neurons and VZV challenge of hiPSC-macrophages. Nevertheless, as discussed above, active herpes zoster lesions contain both macrophages and lymphocytes, and as such our proposed bi-cellular co-culture model may need to be extended with the intention of studying neuron-macrophage-lymphocyte interactions in order to more closely resemble the *in vivo* herpes zoster environment, and eventually demonstrate the interaction of innate and adoptive immune cells to be successful in counteracting VZV immune-evasive strategies.

## Conclusion

In this study we established an autologous co-culture model of hiPSC-neurons and hiPSC-macrophages and have shown that even though hiPSC-macrophages are functionally competent, they are unable as a stand-alone population to control an ongoing VZV infection in hiPSC-neurons. Based on our RNA-Seq analysis, we confirm that hiPSC-neurons are largely irresponsive to an ongoing VZV infection, while hiPSC-macrophages do respond upon VZV challenge. Future research now needs to determine the biological relevance and/or consequences of this VZV-induced hiPSC-macrophage response in more complex neuro-immune co-culture models of VZV infection.

## Data availability statement

The datasets presented in this study can be found in online repositories. The names of the repository/repositories and accession number(s) can be found here: GSE228390 (GEO).

## Author contributions

Conceptualization: PP, BO, PD, TB-H, EB, and MB; methodology: PP, BO, PD, KL, PM, AB, MA-R, and CM; investigation: TB-H with help of EB, JG, PM, MB, JS, TC, HR, LD, JS, EB, AB, and PR; formal analysis: TB-H, JG, and EB; writing—original draft: EB, JG, and PP; writing—review and editing: PP, BO, PD, PM, AB, ML, CS-D, CM, and KL; funding acquisition: PP, BO, PD, MA-R, CM, and KL; resources: ML and CS-D; supervision: PP, BO, and PD. All authors contributed to the article and approved the submitted version.

## Supplementary material

Supplementary File 1 annotation list Nanostring Host Response panel.

Supplementary File 2 DEG and pathway analysis hiPSC-neurons.

Supplementary File 3 DEG and pathway analysis hiPSC-macrophages.
