# Supplementary material for: Lack of strong innate immune reactivity renders macrophages alone unable to control productive Varicella-Zoster Virus infection in an isogenic human iPSC-derived neuronal co-culture model
Source: Front Immunol. 2023 May 23;14:1177245. doi: 10.3389/fimmu.2023.1177245 (PMC10241998; doi:10.3389/fimmu.2023.1177245)

**Figure S1 – Manual pre-gating strategy for mass cytometry.**

Beads are discarded by selecting cell events only. Next, doublets are excluded using Gaussian parameters (Residual, Center, Offset, Width). After that, intact cells and singlets are selected and live cells, low in CisPt, are taken for further analysis.


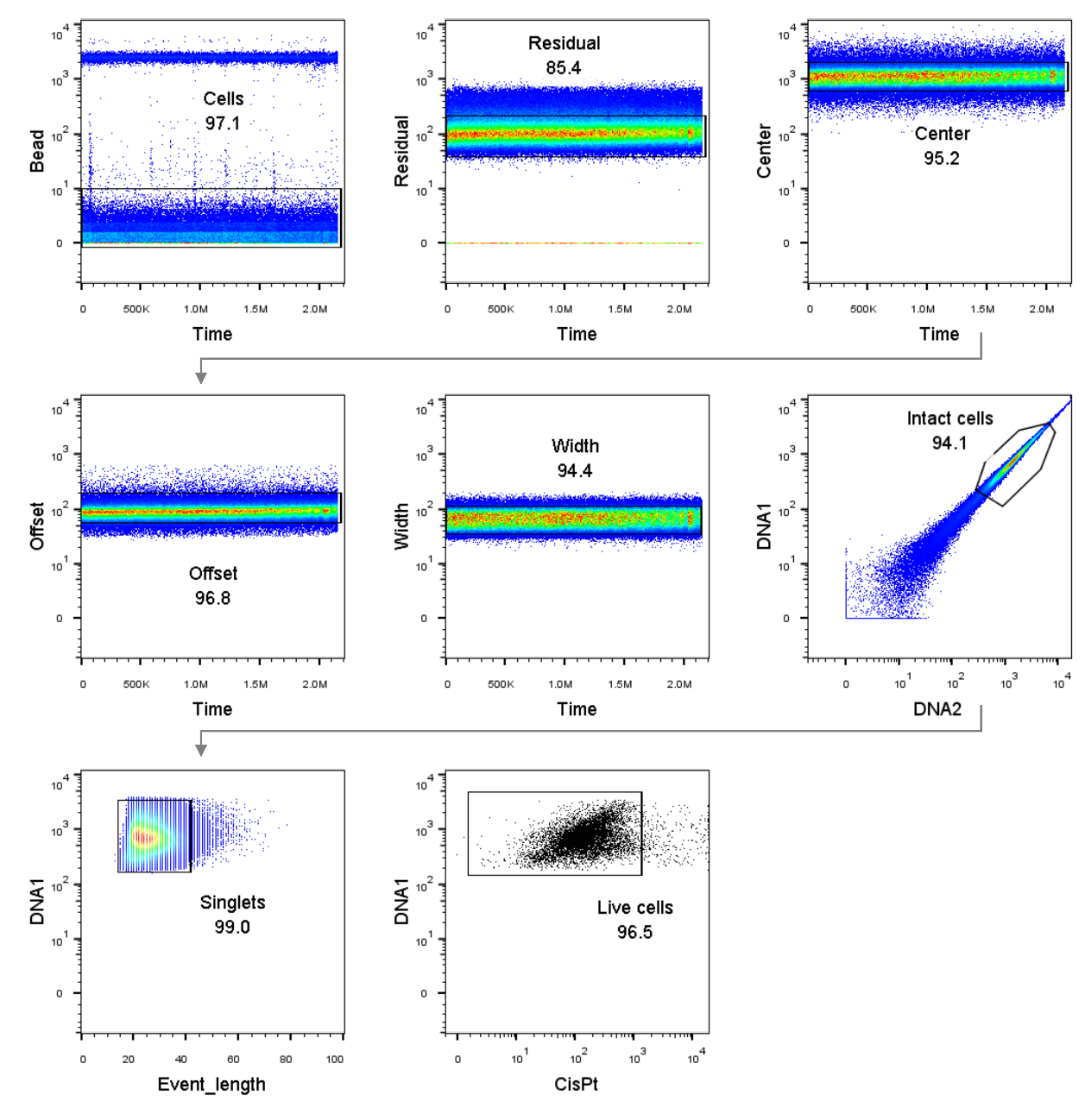

Supplement: Supplementary File 1 — annotation list Nanostring Host Response panel. [file DataSheet_1.docx]
